# *Escherichia coli* strain INF32/16/A: Dataset of raw reads and assembled draft genome

**DOI:** 10.1016/j.dib.2021.107640

**Published:** 2021-11-26

**Authors:** Shuhaila Mat-Sharani, Suhaila Sulaiman, Nik Yusnoraini Yusof

**Affiliations:** a School of Health Sciences, Universiti Sains Malaysia, Kubang Kerian, Kelantan 16150, Malaysia; b FGV R&D Sdn. Bhd., FGV Innovation Centre, PT 23417 Lengkuk Teknologi, Bandar Enstek, Negeri Sembilan 71760, Malaysia; c Institute for Research in Molecular Medicine (INFORMM), Health Campus, Universiti Sains Malaysia, Kubang Kerian, Kelantan, Malaysia

**Keywords:** *Escherichia coli*, Genome sequencing, Pathogenic, Extended-spectrum beta-lactamase

## Abstract

*Escherichia coli* strain INF32/16/A is a gram-negative bacteria which is an extended-spectrum beta-lactamases (ESBL). ESBL is an enzyme that is produced by bacteria to become resistant to existing antibiotic such as extended-spectrum penicillin, cephalosporins, and have been threatening the ability to treat an infection. Therefore, genome analysis will provide an insight of how this bacteria able to evolve and the information obtained will able to facilitate in designing new antibiotics. The genome of *E. coli* strain was sequenced using Illumina MiSeq and raw genome sequence have been submitted into NCBI SRA database (SRR15334628) under Bioproject accession number PRJNA726861.

## Specifications Table

**Table:** 

Subject	Health and medical sciences
Specific subject area	Microbiology and genomics. Genome sequencing of pathogenic bacteria by using next generation sequencing approach.
Type of data	Table Figure Raw reads of sequenced genome Assembled draft genome of *E. coli* strain INF32/16/A
How data were acquired	Paired-end reads of extended spectrum beta lactamase (ESBL)-producing *E. coli* strain INF32/16/A genome were sequenced using Illumina MiSeq.
Data format	Raw and analyzed.
Parameters for data collection	Genomic DNA from pure culture. 10 µg/ng of DNA was utilized for a 251 bp paired-end sequencing library using an Illumina paired-end DNA sample preparation kit.
Description of data collection	Whole genome sequencing performed by Illumina MiSeq system. Raw reads were trimmed using BBDuk (BBTools v36) and assembled using SPAdes v3.9.0. The scaffolding was conducted using Medusa v1.6. The genome completeness of the assembled genome was assessed using BUSCO tool.
Data source location	Institution: Institute for Research in Molecular Medicine (INFORMM) City/Town/Region: Kubang Kerian, Kelantan Country: Malaysia Latitude and longitude for collected samples/data: 6.10 N 102.28 E
Data accessibility	The data is hosted on a public repository. Bioproject: https://www.ncbi.nlm.nih.gov/bioproject/PRJNA726861 Biosample: https://www.ncbi.nlm.nih.gov/biosample/SAMN18971244 NCBI GenBank Accession Number: NZ_JAGWDO010000000.1 https://www.ncbi.nlm.nih.gov/nuccore/2035338809 Repository name: NCBI SRA database Data identification number: SRR15334628 Direct URL to data: https://trace.ncbi.nlm.nih.gov/Traces/sra/?run=SRR15334628

## Value of the Data

• The draft genome data of ESBL-producing *E. coli* strain from Malaysia could contribute fundamental knowledge of the emergence of ESBL-type.
• The data is crucial as it will be benefited researchers, medical and health sector to gain information on antimicrobial resistance genes of ESBL-producing *E. coli* strains from Malaysia.
• Whole genome sequence data from ESBL strain would be useful for comparative genomic analysis *E. coli* strains with other types of ESBL genes isolated in different countries.
• By unravelling the genome of this strain, the data may be leveraged by researchers to plan and design new antibiotics targeting the homologous pathogenic bacteria for better future management on emergence of new resistant strains.

## 1 Data Description

**Fig. 1:**
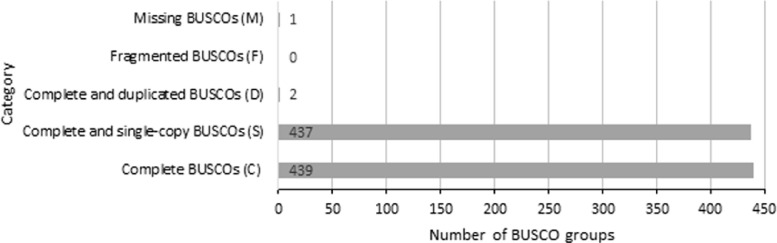
Genome completeness of the assembled genome of *E. coli* strain INF32/16/A by using BUSCO tool with enterobacterales_odb10 lineage.

**Fig. 2:**
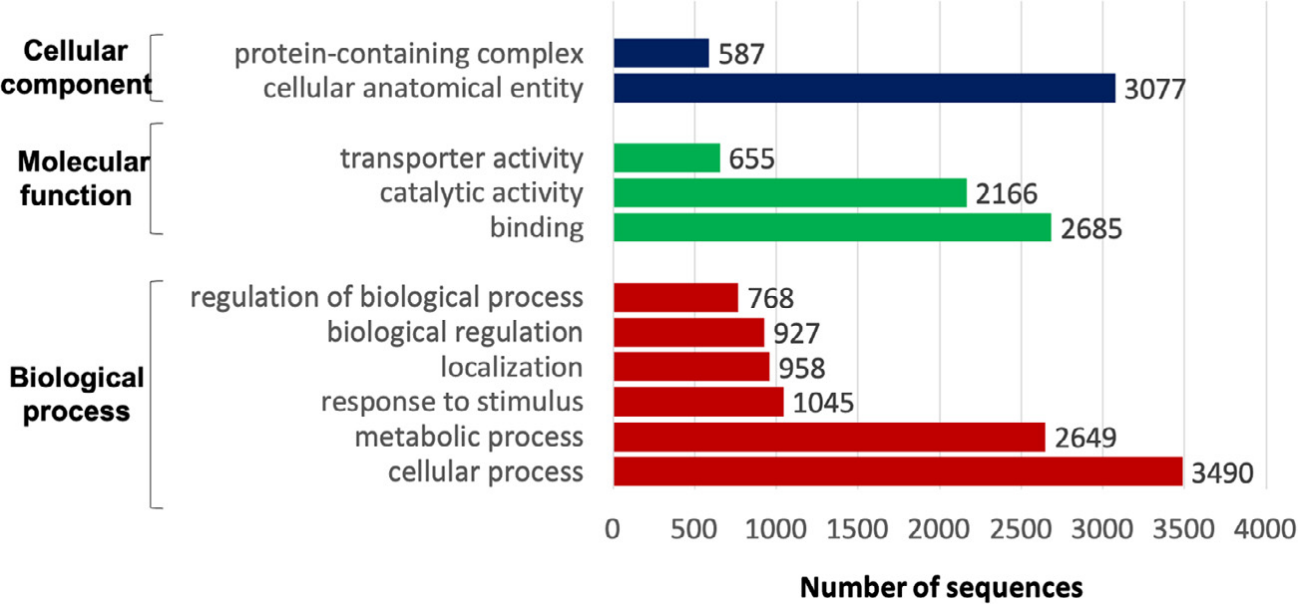
Functional classification based on Gene Ontology for 99.2% of predicted gene models from Prodigal.

This data consists of raw reads of the *E. coli* strain INF32/16/A genome that was sequenced via Illumina MiSeq technology [1]. The paired-end data sets were named as 32-16-A_R1.fastq and 32-16-A_R2.fastq. Here, we report the pre-processing of the raw reads, assembly data statistics, assembled genome completeness and similarity search of the assembled genome with a curated public database. A total of 1,592,134 raw reads (consolidated from a paired-end dataset) were generated from the genome sequencing of *E. coli* strain INF32/16/A, that resulted into 381,590,477 total bases (Table 1). The reads were then pre-processed to filter out reads with low-quality, short, and adapter sequences, that accounts into 53.29% of clean reads. The clean reads were successfully assembled into 97 scaffolds with the longest scaffold being the same as N50 scaffold length of 3,201,741 bases (Table 2). The assembled genome size is 5,212,612 at 74 × sequence coverage with 50.32% GC content. This genome has 4771 protein coding sequences and 313 (9 rRNA, 69 tRNA, 6 ncRNA and 229 pseudo genes) non-coding sequences. The completeness of the genome assembly was evaluated by using Benchmarking Universal Single-Copy Orthologs (BUSCO) [2] with the lineage of enterobacterales_odb10, showing a 99.8% of complete BUSCOs found in the assembled data (Fig. 1). Of 4923 predicted genes by Prodigal [3], homology-based search indicated that 92.2% of them had homology with known curated proteins in Swiss-Prot database [4] with annotated functions in Gene Ontology (Fig. 2).

**Table 1:** Statistics of the pre-processing data of the genome reads containing forward (32-16-A_R1.fastq) and reverse (32-16-A_R2.fastq) reads.

Sample Name	R1	R2	Total
Total Raw Reads	796,067	796,067	1,592,134
Total Raw Reads Bases	190,618,311	190,972,166	381,590,477
Total Clean Reads	424,248	424,248	848,496
Total Clean Reads Bases	85,679,126	57,343,443	143,022,569
Clean Reads (%)	53.29	53.29	53.29 (average)
GC Content Clean Reads (%)	50	51	-

**Table 2:** The main assembly statistics of the Medusa-assembled draft genome of *E. coli* strain INF32/16/A.

Attributes	Value
Number of scaffolds	97
Total size of scaffolds	5,212,612
Longest scaffold	3,201,741
Shortest scaffold	220
Number of scaffolds > 1K nt	62 (63.9%)
Number of scaffolds > 10K nt	18 (18.6%)
Number of scaffolds > 100K nt	4 (4.1%)
Number of scaffolds > 1M nt	1 (1.0%)
Number of scaffolds > 10M nt	0 (0.0%)
Mean scaffold size	53,738
Median scaffold size	1,667
N50 scaffold length	3,201,741
L50 scaffold count	1
GC Content	50.32%

## 2 Experimental Design, Materials and Methods

### 2.1 Material

The clinical isolate of ESBL *E. coli* strain was drawn from a patient's blood with nosocomial infection at Hospital Universiti Sains Malaysia. The clinical sample was collected and confirmed according to the previously described [5]. Briefly, the blood sample was cultured in Bactec 9240 blood culture system (Becton, Dickinson, USA). Then, the strain was tested with gram staining and biochemical techniques prior to evaluate the ESBL screening using Clinical and Laboratory Standards Institute (CLSI) [6]. It was then subcultured into nutrient broth and incubated in a shaking incubator overnight at 37 °C and 200 rpm. The DNA was extracted and examined with 2% electrophoresis gel and NanoDrop® ND-1000 Spectrophotometer (Thermo Scientific) for the purity quantification.

### 2.2 Genome sequencing

A total of 2.5 µg of DNA was used to prepare a 251 bp paired-end sequencing library using an Illumina paired-end DNA sample preparation kit. The quality of the library was assessed by real time PCR before continuing to cluster generation. Sequencing was performed using one lanes of Illumina MiSeq paired-end flow cell using 500 cycles to produce 2 × 251 bp paired-end reads.

### 2.3 Quality assessment and reads pre-processing

During preparation for sequencing, the bacteria genomic DNA fragments were attached to the sequencing adapters, which contains the anchoring site of the sequencing primers. Thus, all sequencing reads were scanned to filter the sequencing adapters to retain the portion containing the bacteria genomic DNA. Besides, trimming was also done for low quality bases (<Q30) and short reads (<50 bp) to ensure clean reads containing high quality reads dataset. The quality assessment of these reads was performed using FASTQC (https://www.bioinformatics.babraham.ac.uk/projects/fastqc/) while the adapter trimming, quality trimming, contaminant filtering and read length filtering was done using BBDuk (BBTools version 36) (http://jgi.doe.gov/data-and-tools/bbtools/). Table 1 shows the pre-processing statistics of the genome reads.

### 2.4 Genome draft assembly

The high-quality reads of Illumina were assembled *de novo* using SPAdes v3.9.0 [7]. These contigs were subjected to scaffolding against the closest reference genomes [5] to produce a draft genome using Medusa (Multi-Draft based Scaffolder) software [8]. The top two hits complete genome from GenBank, *E. coli* strain AR-0427 (CP044148.1) and *E. coli* strain AR216.2b (CP043942), were used to construct the draft genome scaffold. The assembly statistics is shown in Table 2. The completeness of assembled draft genome was assessed by using BUSCO on a LINUX server. Bacteria dataset of enterobacterales_odb10 was used as its single-copy orthologs database (Fig. 1). The predicted genes by Prodigal that was generated by BUSCO analysis were searched against Swiss-Prot database to see the similarity of the assembled sequence. The similarity search shows about 92.2% of the predicted genes were similar to the manually curated protein database. These known genes were associated to different gene ontology classes, with the highest in biological process, molecular function and cellular component being cellular process (3490), binding (2,685) and cellular anatomical entity (3,077) (Fig. 2).

## Ethics Statement

The study protocol was approved by the ethics committee of the Universiti Sains Malaysia (USM/JEPeM/20030152).

## CRediT authorship contribution statement

**Shuhaila Mat-Sharani:** Software, Formal analysis, Data curation, Writing – review & editing. **Suhaila Sulaiman:** Conceptualization, Software, Formal analysis, Data curation, Writing – original draft. **Nik Yusnoraini Yusof:** Conceptualization, Software, Methodology, Resources, Writing – review & editing, Supervision, Funding acquisition.

## Declaration of Competing Interest

The authors declare that they have no known competing financial interests or personal relationships which have, or could be perceived to have, influenced the work reported in this article.
